# The human nucleophosmin 1 mutation A inhibits myeloid differentiation of leukemia cells by modulating miR-10b

**DOI:** 10.18632/oncotarget.12216

**Published:** 2016-09-23

**Authors:** Qin Zou, Shi Tan, Zailin Yang, Juan Wang, Jingrong Xian, Shuaishuai Zhang, Hongjun Jin, Liyuan Yang, Lu Wang, Ling Zhang

**Affiliations:** ^1^ Key Laboratory of Laboratory Medical Diagnostics Designated by the Ministry of Education, College of Laboratory Medicine, Chongqing Medical University, Chongqing, China; ^2^ Department of Clinical Laboratory, Chongqing Health Center for Women and Children, Chongqing, China; ^3^ Center for Hematology, Southwest Hospital, Third Military Medical University, Chongqing, China; ^4^ Children's Medical Laboratory Diagnosis Center, Qilu Children's Hospital of Shandong University, Jinan, China

**Keywords:** nucleophosmin 1, mutation, microRNA, leukemia, differentiation

## Abstract

Mutations in the nucleophosmin 1 (NPM1) gene are the most frequent genetic alteration in acute myeloid leukemia (AML). Here, we showed that enforced expression of NPM1 mutation type A (NPM1-mA) inhibits myeloid differentiation of leukemia cells, whereas knockdown of NPM1-mA has the opposite effect. Our analyses of normal karyotype AML samples from The Cancer Genome Atlas (TCGA) dataset revealed that miR-10b is commonly overexpressed in NPM1-mutated AMLs. We also found high expression of miR-10b in primary NPM1-mutated AML blasts and NPM1-mA positive OCI-AML3 cells. In addition, NPM1-mA knockdown enhanced myeloid differentiation, while induced expression of miR-10b reversed this effect. Finally, we showed that KLF4 is downregulated in NPM1-mutated AMLs. These results demonstrated that miR-10b exerts its effects by repressing the translation of KLF4 and that NPM1-mA inhibits myeloid differentiation through the miR-10b/KLF4 axis. This sheds new light on the effect of NPM1 mutations' on leukemogenesis.

## INTRODUCTION

Acute myeloid leukemia (AML) arises from multiple, sequential genetic alterations. Mutations in the nucleophosmin 1 (NPM1) gene are the most common, accounting for approximately one third of all adult *de novo* AML, especially in patients with normal karyotype AML (NK-AML) [1]. NPM1 is an abundant, multifunctional nucleolar protein with nuclear-cytoplasmic shuttling activity [2] and plays multiple roles in cell growth and proliferation [3, 4]. AML carrying *NPM1* mutations is a distinct AML entity in the 2016 World Health Organization (WHO) classification of myeloid neoplasms because of its distinct biological and clinical features [5]. Approximately 60 different types of *NPM1* mutations have been found, the most common being the type A mutation (*NPM1-mA*) with a four base (TCTG) insertion at exon 12 [4]. All molecular variants of NPM1 mutation result in changes at the C terminus of *NPM1* that interfere with its nucleo-cytoplasmic traffic, giving rise to the aberrant cytoplasm-dislocated mutant NPM1 (NPM1c+) protein [4, 6]. The genetic events underlying AML pathogenesis could fall into two broad groups. One comprises mutations that enhance proliferation and/or survival of hematopoietic progenitors; the other comprises mutations that perturb hematopoietic differentiation and/or aberrant acquisition of self-renewal [7]. It has been shown that intracellular NPM1 mutants can mediate cell cycle induction, apoptosis resistance and proliferation promotion by binding to other nuclear proteins and delocalizing them into the cytoplasm, including the p14ARF [8], PTEN [9] and Fbw7γ [10]. This suggests that NPM1 mutants promote leukemogenesis. Clinical evidence has pointed to NPM1-mutated AML exhibiting limited differentiation [11]. Leong *et al.* [12] confirmed that *NPM1* mutation dysregulated myeloid differentiation through inhibition of caspase 6 and 8. Furthermore, aberrant expression of miRNAs in AML has been reported to be a critical player in malignant myeloid differentiation [13, 14]. However, little is known about the specific impact of *NPM1* mutations and miRNA expression on leukemia cell differentiation.

MicroRNAs (miRNAs) are a class of small noncoding RNAs that negatively regulate gene expression at posttranscriptional levels [15]. Aberrant miRNA expression correlates with specific cytogenetic and clinical prognosis [16, 17]. Recently, a unique miRNA signature has been identified and thought to be associated with AML patients harboring *NPM1* mutation including the strong upregulation of miR-10b [18]. MiR-10b is embedded in the homeobox (HOX) gene clusters [19]. Studies revealed that miR-10b could regulate metastasis, invasion and cell differentiation through targeting HOXD10 [20], Kruppel-like factor KLF4 [21] and nuclear receptor corepressor 2 (NCOR2) [22]. Within the hematopoietic system, the KLFs family regulates cellular development, growth, and differentiation [23]. Loss- and gain-of-function experiments in leukemia cells demostrated that KLF4 controls monocyte differentiation [24]. However, the specific role of miR-10b in NPM1-mutated leukemia has not been fully elucidated.

In light of these findings, we hypothesized that NPM1 mutations might inhibit myeloid differentiation of leukemia cells. Herein, we confirmed that NPM1 mutations inhibited myeloid differentiation, mediated by miR-10b and its binding target KLF4.

## RESULTS

### NPM1-mA inhibits PMA-induced myeloid differentiation of leukemia cells

**Figure 1:**
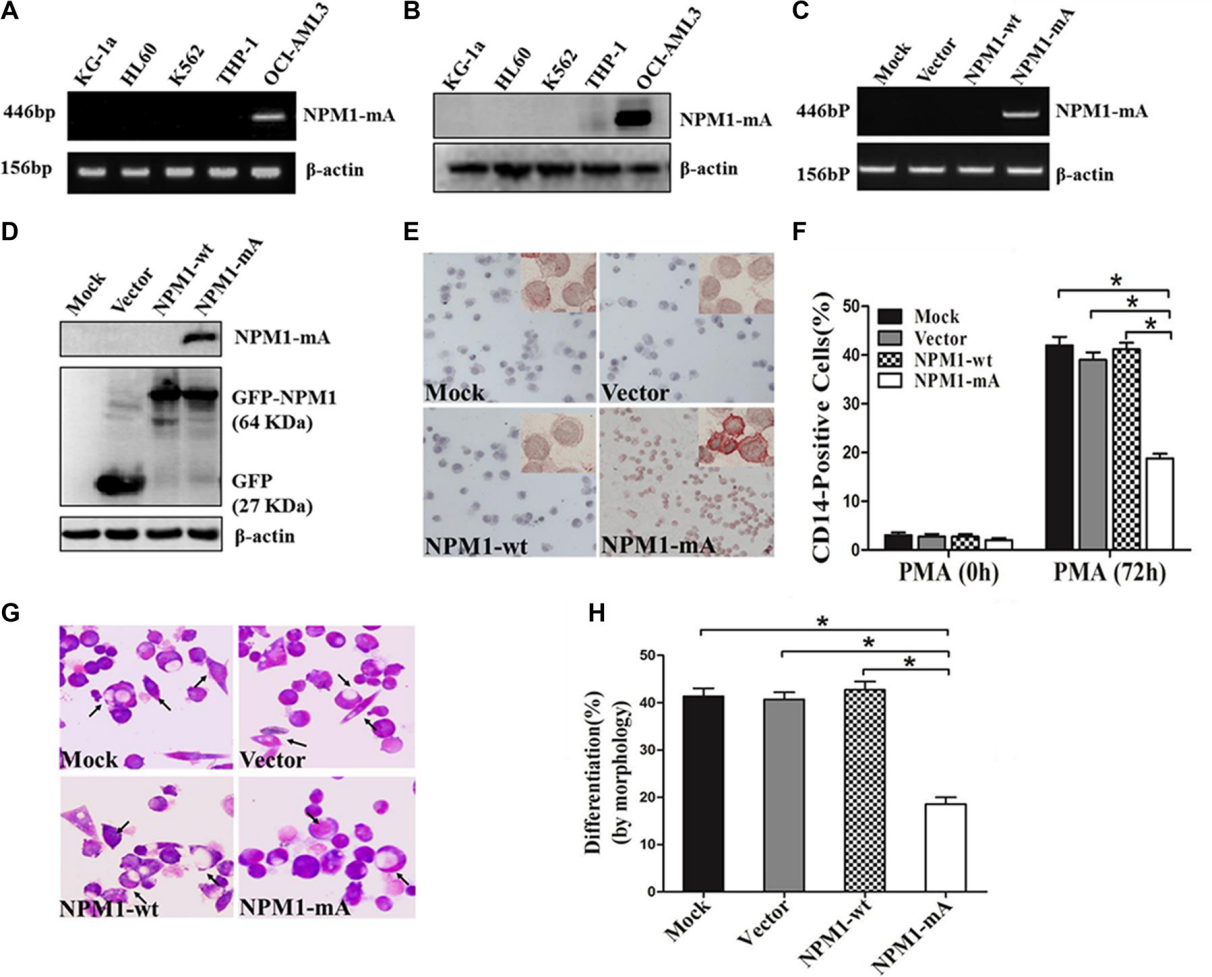
Overexpression of NPM1-mA inhibits myeloid differentiation of THP-1 cells (**A**–**B**) RT-PCR and western blot showing the absence of NPM1-mA mRNA and protein expression in KG-1a, HL60, K562 and THP-1 cells. OCI-AML3 cells were included as positive control. β-actin served as the loading controls. (**C**–**D**) NPM1-mA expression in THP-1 cells transfected with the recombinant plasmids expressing NPM1-mA, NPM1-wt or empty vector, measured by RT-PCR and western blot. (**E**) Representative results of cytoplasm-dislocated NPM1 mutant protein detected by immunocytochemistry staining (APAAP) in NPMc+ cells of the NPM1-mA group (×200). (**F**) The expression of CD14 in transfected THP-1 cells followed by PMA induction for 72 h was determined by flow cytometry. (**G**) Representative Wright-Giemsa staining images of transfected cells induced by PMA for 72 h. The arrows point to differentiated cells. (**H**) Percentage of differentiated cells determined by counting at least 200 cells on the slides under a light microscope. Three independent experiments were performed. *P < 0.05.

To investigate the role of NPM1-mA on differentiation of leukemia cells, We first confirmed that, in contrast to AML cell line OCI-AML3 naturally carrying NPM1-mA [25], the AML cell lines KG-1a, HL60, K562 and THP-1 lacked NPM1-mA expression (Figure 1A–1B). THP-1 cells derived from human monocytic leukemia were stably transfected with the recombinant plasmids expressing NPM1-mA, NPM1-wt or empty vector. These results showed that the cells in the NPM1-mA group expressed NPM1-mA mRNA and protein (Figure 1C–1D). Additionally, immunocytochemistry staining results demonstrated that the NPM1 mutant protein remained restricted to the cytoplasm in the NPM1-mA group, as shown by the presence of red precipitate particles (Figure 1E). These data indicated that leukemia cells with stable NPM1-mA expression were successfully constructed. Consistent with previous reports revealing the capacity of PMA to induce myeloid differentiation in THP-1 cells, PMA treatment also increased the expression of the myeloid-specific surface markers CD14 in THP-1 cells [26]. Compared with the three control groups, lower percentages of CD14-positive cells were observed in the NPM1-mA group as determined by flow cytometry (Figure 1F). In addition, Wright-Giemsa staining demonstrated that cells in the NPM1-mA group exhibited immature morphological features (increase in the ratio of nuclear/cytoplasmic fractions, and round nuclei) (Figure 1G) and a lower proportion of differentiated cells (*P* < 0.05, Figure 1H). These data indicated that overexpression of NPM1-mA impaired PMA-induced myeloid differentiation of leukemia cells.

**Figure 2:**
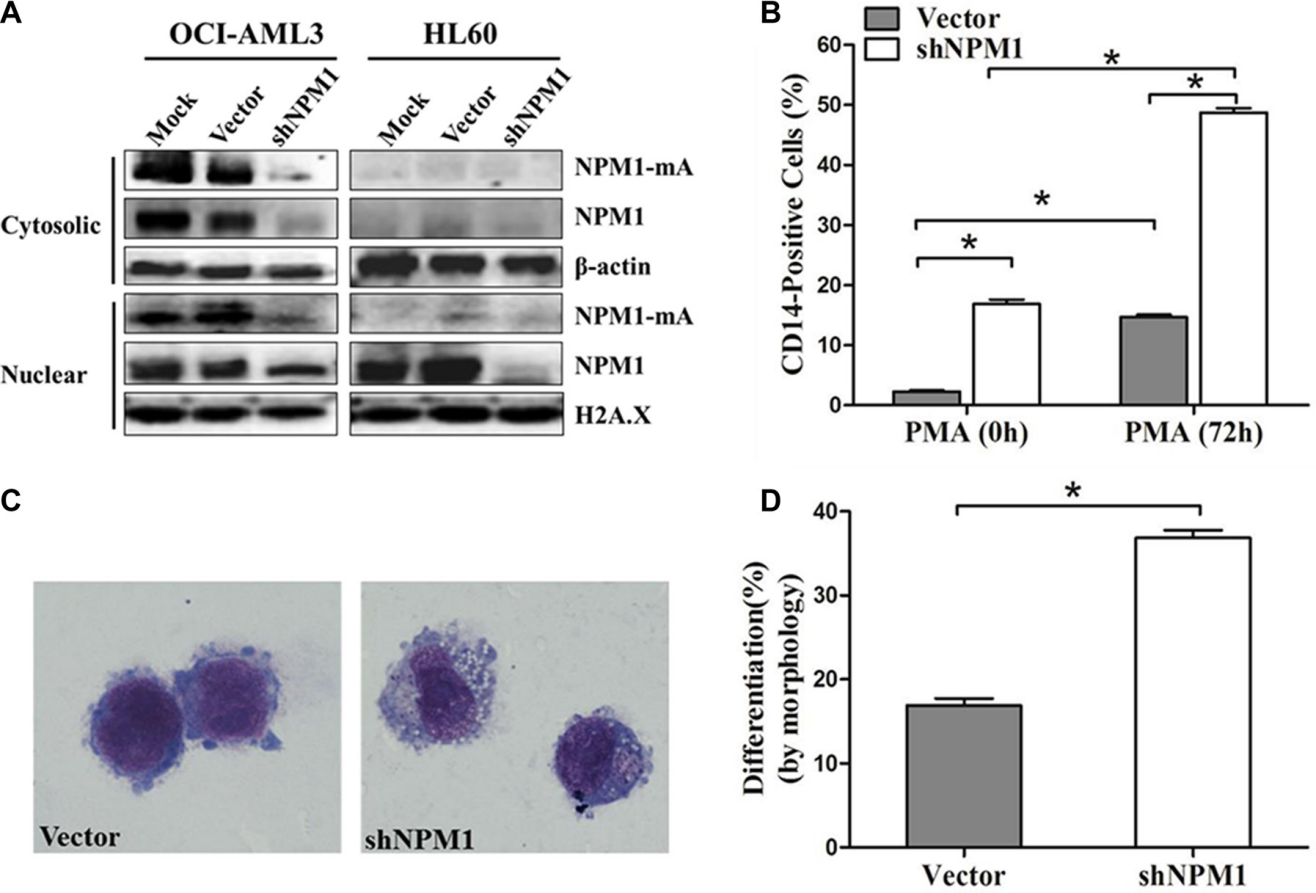
Knockdown of NPM1-mA enhances myeloid differentiation of OCI-AML3 cells (**A**) Western blot of NPM1-mA and NPM1 in the nuclear and cytosolic fractions of OCI-AML3 infected with empty vector or the shNPM1 lentivirus, HL60 cells served as control. β-actin (cytosolic) and H2A.X (nuclear) served as the loading controls. (**B**) Flow cytometry showing the percentage of the CD14-positive cells in infected OCI-AML3 cells following PMA treatment for 72 h. (**C**) Representative Wright-Giemsa staining image of infected OCI-AML3 cells followed by PMA treatment. (**D**) Percentage of differentiated cells determined by counting at least 200 cells on the slides under a light microscope. Three independent experiments were performed. *P < 0.05.

We then tested the effects of NPM1-mA depletion on myeloid differentiation in leukemia cells. OCI-AML3 cells were infected with shRNA lentivirus targeting *NPM1*, HL60 cells only expressing wild type NPM1 were used as control. As reported previously by others, treatment with shRNA targeting *NPM1* depleted NPM1-mA levels in both the cytoplasmic and nuclear fractions of OCI-AML3 cells, and attenuated NPM1 in OCI-AML3 and HL60 cells (Figure 2A) [27]. We then induced the established OCI-AML3 cells by PMA for 72 h. A higher percentage of CD14-positive cells were observed in the shNPM1 group compared with the vector group (*P* < 0.05, Figure 2B). Furthermore, cells in the shNPM1 group exhibited morphological features of differentiation (clear cytoplasm and cytoplasmic vacuoles; Figure 2C) and a higher proportion of differentiated cells (*P* < 0.05, Figure 2D). These data indicated that knockdown of NPM1-mA enhanced PMA-induced myeloid differentiation of leukemia cells.

### MiR-10b is upregulated in NPM1-mutated AML

**Figure 3:**
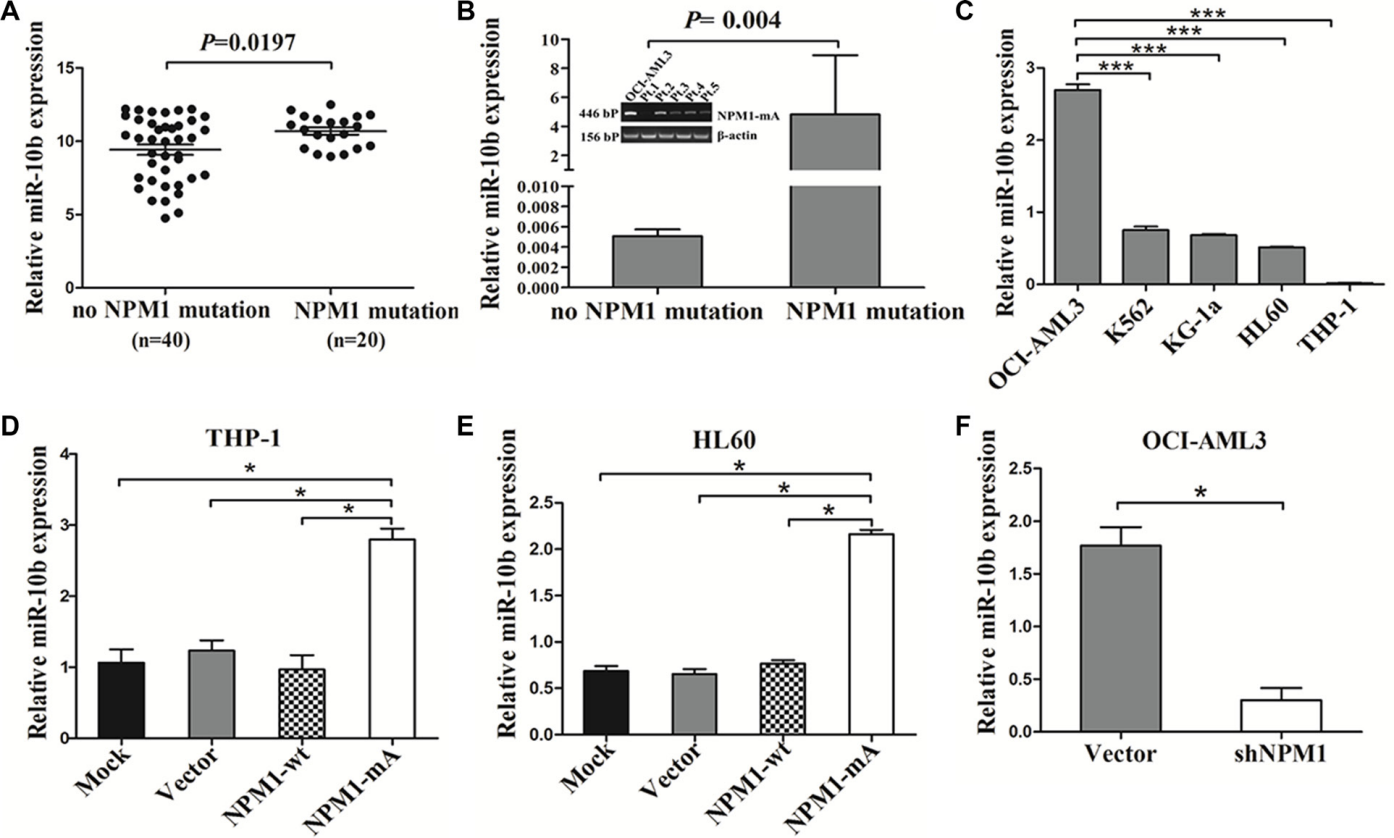
MiR-10b is upregulated in NPM1-mutated leukemia cells (**A**) *t* test using RNA-Seq mRNA expression data from the TCGA database to compare relative miR-10b expression between NK-AML patients with (*n* = 20) and without *NPM1* mutation (*n* = 40). (**B**) Levels of miR-10b in 4 primary NPM1-mutated AML cases assessed by qRT-PCR, and compared with 8 NPM1-unmutated AMLs. (**C**) Levels of miR-10b in 5 myeloid leukemia cell lines. Changes in miR-10b levels in (**D**) THP-1 and (**E**) HL60 cells following NPM1-mA overexpression, and. (**F**) in OCI-AML3 cells following NPM1-mA knockdown. Three independent experiments were performed. *P < 0.05, ***P < 0.001.

A previous study documented that a unique miRNA signature was associated with NPM1-mutated AML and miR-10b expression in clearly differentiated NPMc+ vs. NPMc- cases [16]. To analyze the levels of miR-10b expression in AML with *NPM1* mutation, we accessed the RNA-Seq data of 60 NK-AML patients from the TCGA AML dataset and found that the expression of miR-10b was higher in the AMLs with *NPM1* mutation compared with those with no *NPM1* mutation (*P* = 0.0197, Figure 3A). Next, we analyzed the levels of miR-10b in primary AML samples by qRT-PCR, and found that miR-10b levels were higher in NPM1-mutated AML compared with those in NPM1-unmutated AML (*P* = 0.004, Figure 3B). A similar result was observed in OCI-AML3 cells naturally carrying *NPM1* mutations (Figure 3C). Next, we analyzed the change in miR-10b levels by gain- and loss of NPM1-mA function experiments. The results revealed that enforced NPM1-mA expression in leukemia cells (THP-1 and HL60) increased miR-10b expression (Figure 3D–3E), whereas silenced NPM1-mA expression in OCI-AML3 cells decreased miR-10b levels (Figure 3F). These results demonstrated that upregulation of miR-10b in NPM1-mutated AML was at least partially due to NPM1-mA expression.

### MiR10b is involved in myeloid differentiation of leukemia cells with NPM1-mA

**Figure 4:**
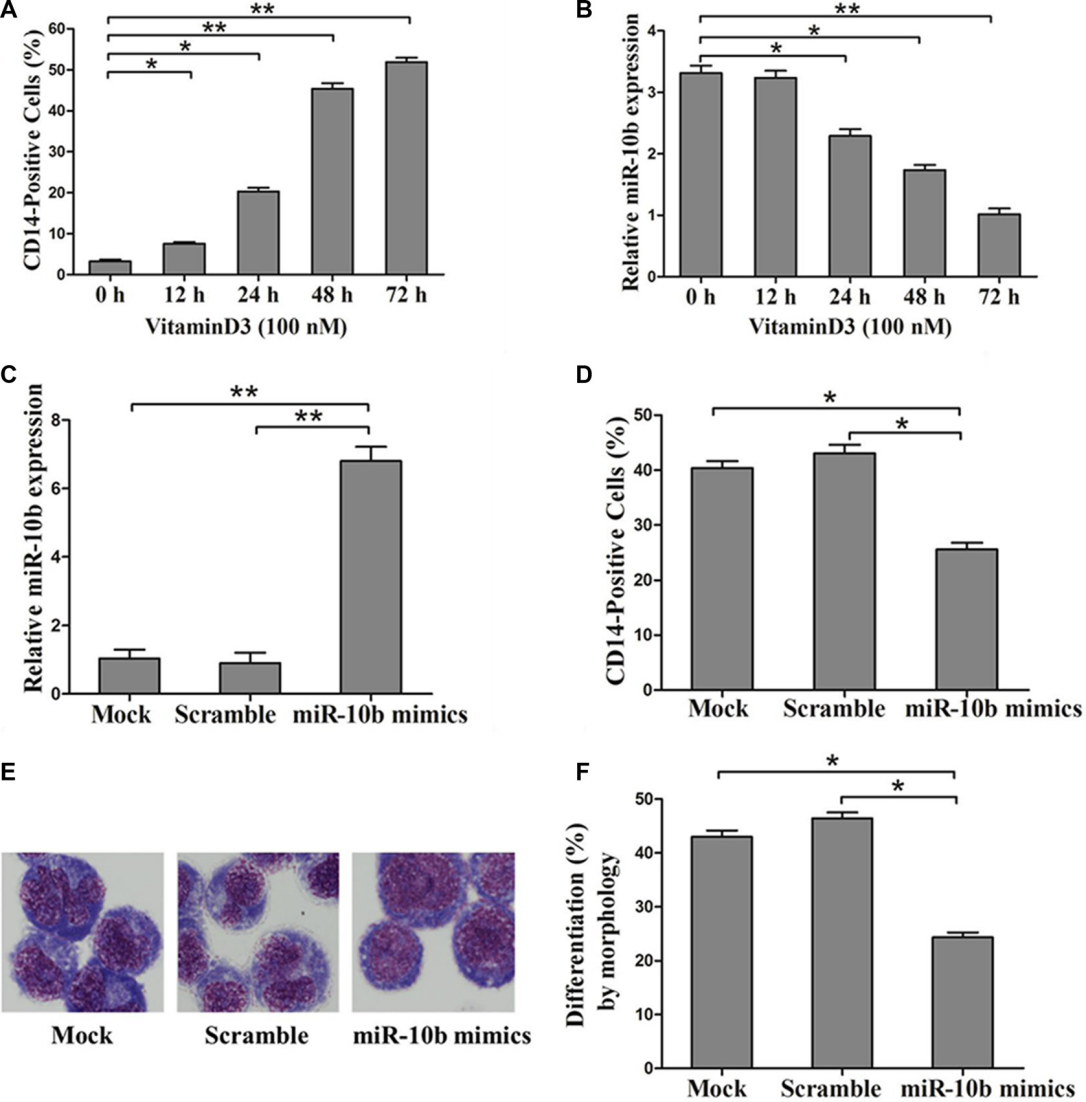
Overexpression of miR-10b inhibits myeloid differentiation of HL60 cells (**A**) Expression of CD14 measured by flow cytometry and (**B**) of miR-10b assessed by qRT-PCR during Vitamin D3-induced differentiation of HL60 cells. (**C**) Expression of miR-10b measured by qRT-PCR and (**D**) of CD14 measured by flow cytometry in HL60 cells transfected with miR-10b scramble or mimics under Vitamin D3 treatment for 72 h. (**E**) Representative Wright-Giemsa staining image of transfected cells treated with Vitamin D3 for 72 h. (**F**) Percentage of differentiated cells determined by counting at least 200 cells under a light microscope. Three independent experiments were performed. *P < 0.05, **P < 0.01.

**Figure 5:**
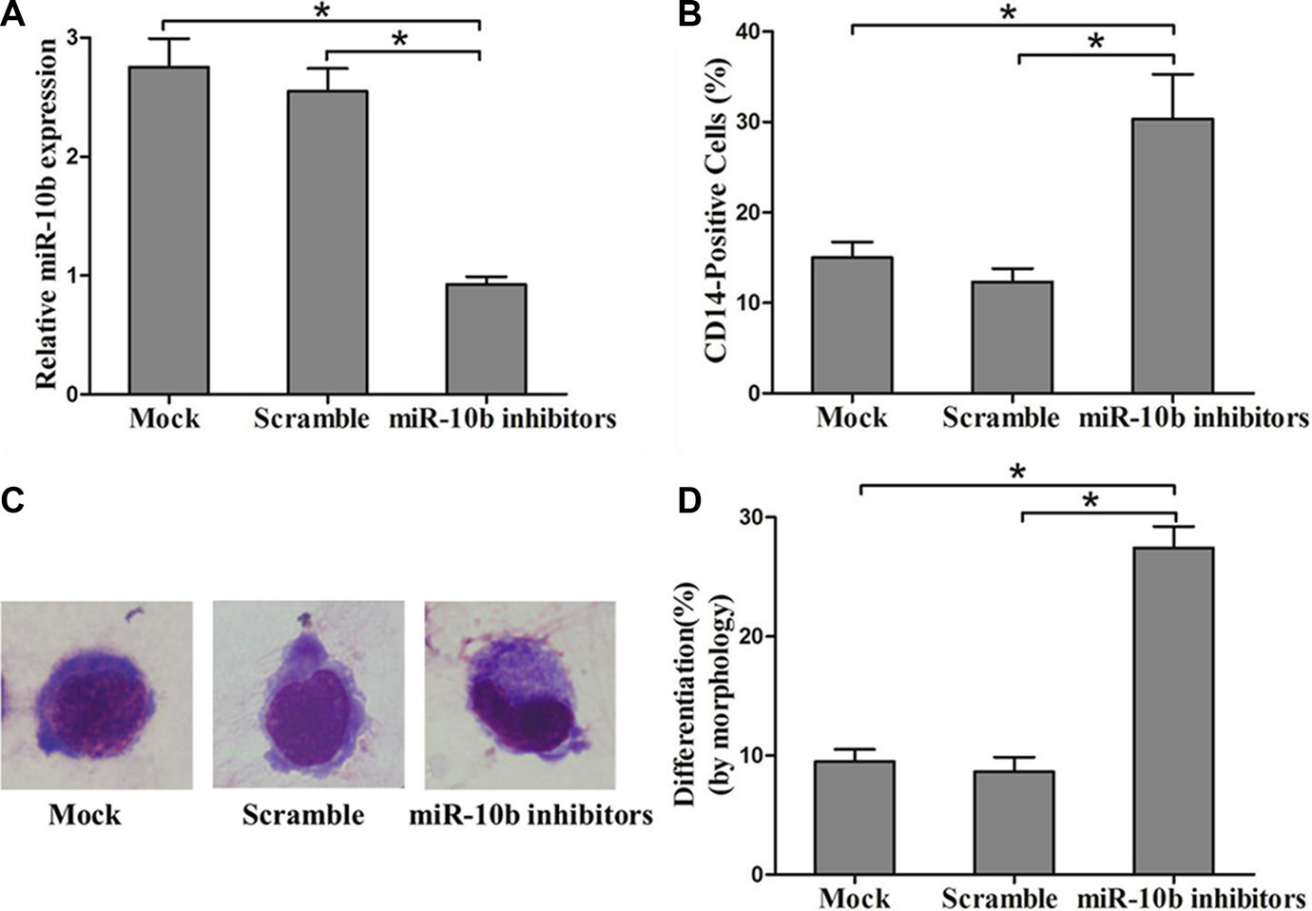
Suppression of miR-10b promotes myeloid differentiation of OCI-AML3 cells (**A**) Levels of miR-10b in OCI-AML3 cells transfected with miR-10b scramble or inhibitors measured by qRT-PCR. (**B**) Expression of CD14 in transfected OCI-AML3 cells under PMA treatment for 72 h as determined by flow cytometry. (**C**) Representative Wright-Giemsa staining image of transfected OCI-AML3 cells under PMA treatment for 72 h. (**D**) Percentage of differentiated cells determined by counting at least 200 cells under a light microscope. Three independent experiments were performed, *P < 0.05.

To examine whether miR-10b is involved in myeloid differentiation of leukemia cells, we used the human myeloid leukemia HL60 cell line on which cell differentiation assays can be readily performed [28]. Vitamin D3, a regent frequently used to induce differentiation of HL60 cells to monocytes was used for further study. As reported previously by others, we found that CD14 expression increased in a time-dependent manner with Vitamin D3 treatment (Figure 4A) [29]. In addition, miR-10b expression decreased over the time course of the Vitamin D3-induced differentiation (Figure 4B). Next, we transfected miR-10b mimics into leukemia cells, followed by Vitamin D3 induction for 72 h. Results from qRT-PCR analysis revealed increased miR-10b expression (Figure 4C). Furthermore, miR-10b upregulation decreased the percentage of CD14-positive cells (Figure 4D) and suppressed cell differentiation (Figure 4E, 4F). Finally, we investigated the effect of miR-10b downregulation on cell differentiation. Antisense inhibitors directed against miR-10b were transfected into OCI-AML3 cells, followed by PMA treatment for 72 h. We found decreased miR-10b expression (Figure 5A), along with an increase in the number of CD14-positive cells (Figure 5B) and differentiated cells (Figure 5C–5D). These data suggested that miR-10b inhibits myeloid differentiation of leukemia cells.

**Figure 6:**
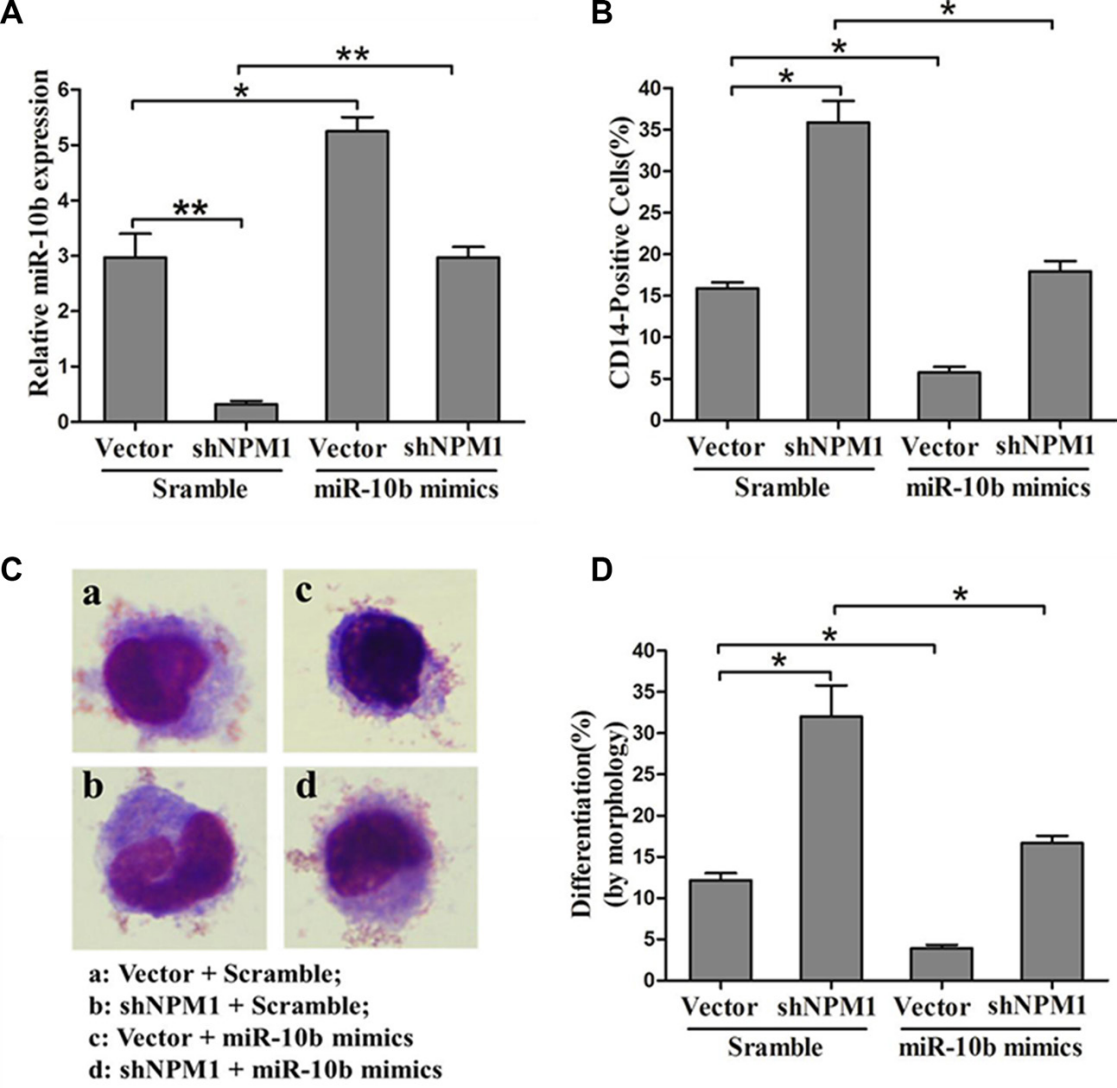
MiR-10b is involved in NPM1-mA-mediated myeloid differentiation of leukemia cells (**A**) Expression of miR-10b measured by qRT-PCR and (**B**) of CD14 measured by flow cytometry in OCI-AML3 cells expressing shNPM1 and transfected with miR-10b scramble or mimics after PMA induction for 72 h. (**C**) Representative Wright-Giemsa staining image of transfected cells. (**D**) Percentage of differentiated cells determined by counting at least 200 cells under a light microscope. Three independent experiments were performed, *P < 0.05, **P < 0.01.

To further explore the role of miR-10b in the inhibition of myeloid differentiation induced by NPM1-mA, we transfected miR-10b mimics or scramble into OCI-AML3 cells stably expressing shNPM1, followed by PMA induction for 72 h. As expected, retransfection with miR-10b mimics alleviated the downregulation of miR-10b resulting from NPM1 shRNA treatment (Figure 6A). Furthermore, forced expression of miR-10b also abolished the enhancement of myeloid differentiation caused by NPM1 knockdown, as revealed by a decrease in the number of CD14-positive cells (Figure 6B) and differentiated cells (Figure 6C–6D). These results demonstrated that miR-10b induces inhibition of myeloid differentiation downstream of NPM1-mA.

### KLF4 is a direct target of miR-10b in AML

**Figure 7:**
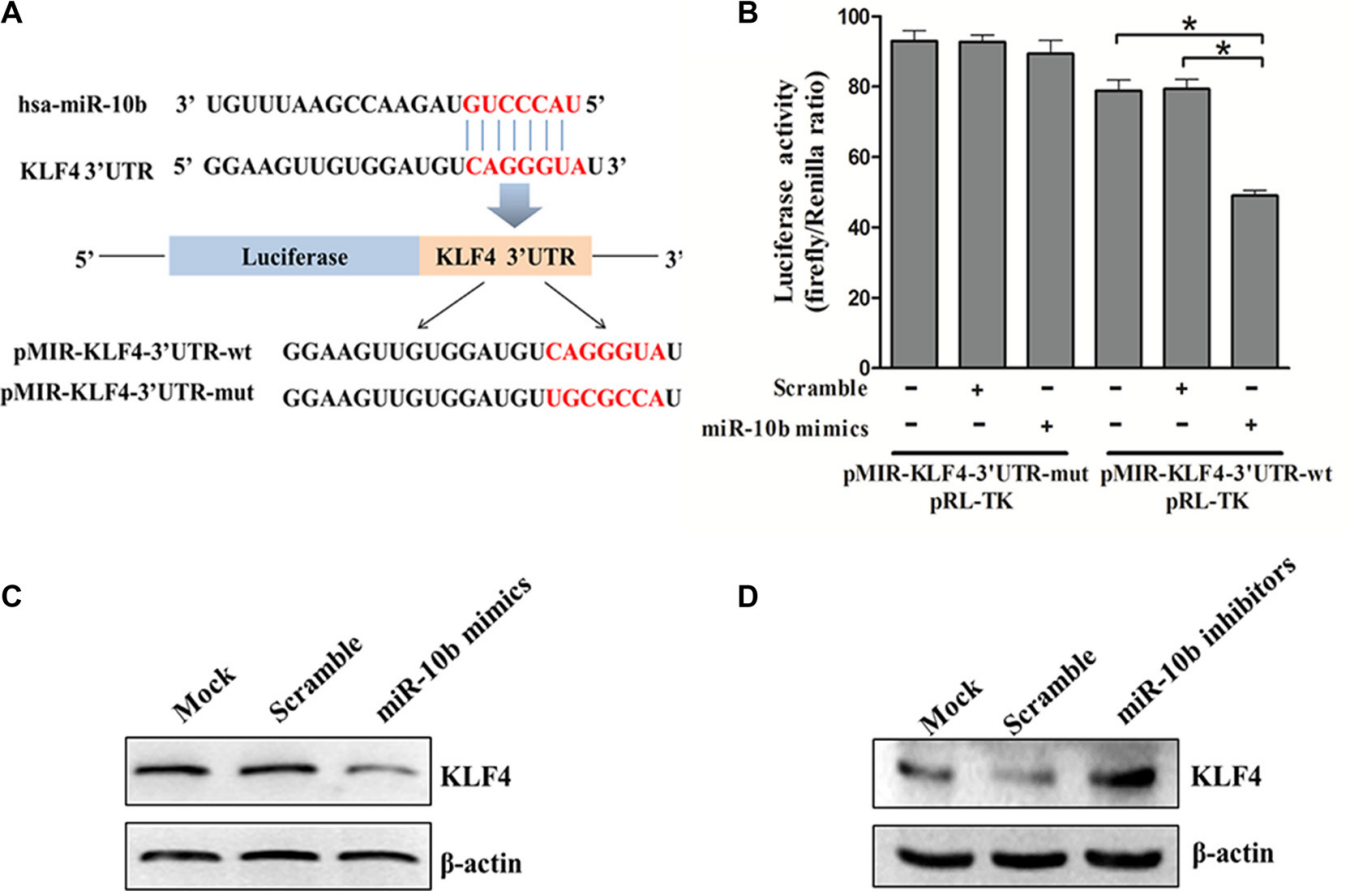
KLF4 is a direct target of miR-10b (**A**) Predicted base pairing between miR-10b and 3′UTR of KLF4 by TargetScan, shown for wild-type (wt) or mutated (mut) 3′UTR of KLF4 inserted into dual-luciferase vector. (**B**) Dual-luciferase analysis of miR-10b mimics/scramble co-transfected with either pMIR-KLF4-3′UTR-wt or pMIR-KLF4-3′UTR-mut into HEK293T cells. KLF4 protein levels in (**C**) HL60 cells and (**D**) OCI-AML3 cells transfected with miR-10b mimics or scramble as measured by western blot (β-actin served as the loading control). Three independent experiments were performed. *P < 0.05.

To understand the mechanisms by which miR-10b inhibited myeloid differentiation, the TargetScan and PicTar search programs were used to help identify miR-10b targets in humans. Among the targets predicted, KLF4 is of particular interest as it promotes myeloid differentiation in leukemia [24]. Bioinformatics analysis revealed that there was a potential miR-10b binding fragment in the 3′-untranslated region (UTR) of the *KLF4* gene. We then cloned that potential miR-10b binding fragment as well as its mutated form into a pMIR-REPROT™ Luciferase reporter vector (Figure 7A). The luciferase reporter assay showed that forced expression of miR-10b suppressed the activity of the luciferase reporter gene containing the wild-type 3′UTR of KLF4 but not that of the one containing the mutated form (Figure 7B). In support of these results, we observed reduced levels of endogenous KLF4 following miR-10b overexpression in HL60 cells (Figure 7C). In contrast, endogenous KLF4 levels increased following miR-10b knockdown in OCI-AML3 cells (Figure 7D). These results demonstrated that KLF4 was a direct target of miR-10b in leukemia cells.

### KLF4 is downregulated in NPM1-mutated AML

**Figure 8:**
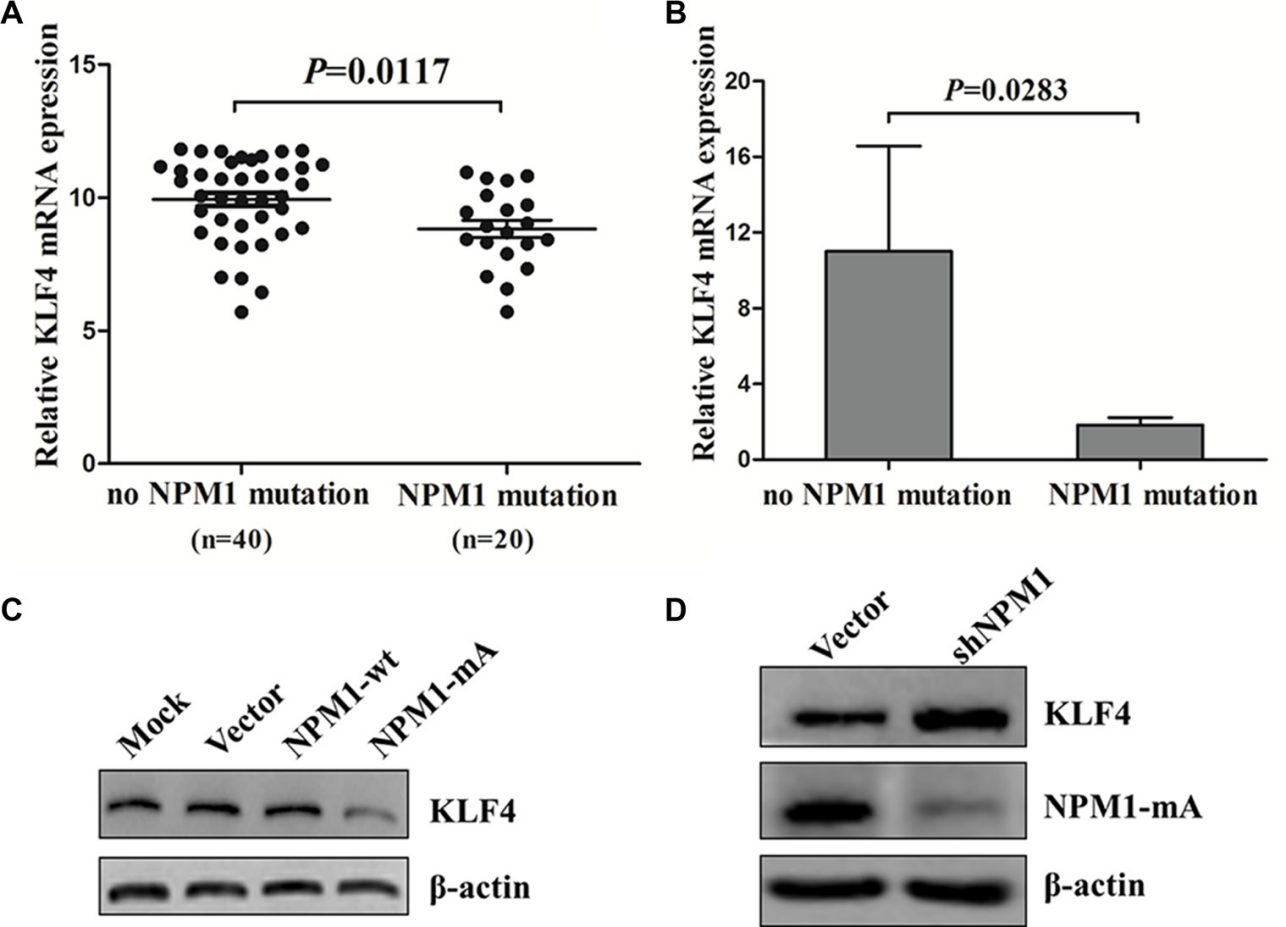
KLF4 is downregulated in NPM1-mutated AML (**A**) Comparison by *t* test of relative *KLF4* mRNA expression in 60 NK-AML patients from the TCGA human AML dataset with (*n* = 20) and without (*n* = 40) NPM1-mutation. (**B**) Expression of *KLF4* mRNA in primary AML patients with (*n* = 4) and without (*n* = 8) NPM1-mutation assessed by qRT-PCR. Levels of KLF4 protein in (**C**) THP-1 cells with NPM1-mA overexpression and (**D**) in OCI-AML3 cells with NPM1-mA knockdown measured by western blot. Three independent experiments were performed.

We also measured KLF4 expression in NPM1-mutated AML. We first evaluated the expression of *KLF4* in the TCGA human AML dataset, and found lower expression of KLF4 mRNA in AML patients with *NPM1* mutation compared with those with no *NPM1* mutation (*P* = 0.0117, Figure 8A). Next, we measured *KLF4* mRNA levels in primary NPM1-mutated AML patients by qRT-PCR. As shown in Figure 8B, *KLF4* mRNA levels are reduced in NPM1-mutated AMLs. To test whether low *KLF4* expression was correlated with *NPM1* mutation *in vitro*, we measured KLF4 protein levels in THP-1 cells overexpressing NPM1-mA. Our results showed that enforced expression of NPM1-mA decreased KLF4 protein levels (Figure 8C). Conversely, silencing of NPM1 in OCI-AML3 cells increased KLF4 protein levels (Figure 8D). These results indicated that KLF4 was downregulated in NPM1-mutated AML.

## DISCUSSION

*NPM1* mutation is the most frequent genetic alteration in AML. Although clinical findings showed that such mutation inhibits cell differentiation, the underlying mechanisms remain largely unknown. Here, we demonstrated that NPM1-mA inhibited myeloid differentiation of leukemia cells *in vitro*, and that the miR-10b/KLF4 axis was involved in this process.

In this study, we first investigated the effect of the NPM1-mA mutation on myeloid differentiation. Since leukemia cells bearing NPM1 mutations frequently show myelomonocytic or monocytic features with dysplasia of two or more cell lineages [30], we selected the THP-1 cell line derived from human monocytic leukemia as a cellular model for experiments. Our data showed that overexpression of NPM1-mA inhibited PMA-induced myeloid differentiation, which was consistent with a previous report demonstrating that the NPM1 mutant could suppress IL-3-mediated myeloid differentiation of cord blood-derived CD34^+^ cells [12]. In addition, we transfected OCI-AML3 cells with shRNA lentivirus targeting NPM1 and found that NPM1 shRNA treatment led to depletion of NPM1-mA in both the cytoplasm and the nucleus. Conversely, NPM1-mA knockdown promoted PMA-induced myeloid differentiation. NPM1 shRNA treatment also lowered NPM1 levels in OCI-AML3 cells, but wild type NPM1 knockdown did not induce differentiation of AML cells [27]. These data suggested that NPM1-mA could inhibit myeloid differentiation of leukemia cells.

Because of the recently discovered miRNA signature of NPM1-mutated AML [18], we hypothesized that miRNA might be involved in the NPM1-mA mediated inhibition of differentiation in leukemia cells. As an RNA-binding protein, NPM1 has been suggested to bind miRNAs, thereby protecting them against degradation [31, 32]. Furthermore, the NPM1 mutant was shown to increase the levels of mature miR-10b [33]. Here, we found high levels of miR-10b in NPM1-mutated AML patients from the TCGA human AML dataset and from analyzing primary AML samples. Our results agreed with previous reports showing that miR-10b was upregulated in NPM1c+ AML samples [18, 34]. More importantly, we found that overexpression of NPM1-mA increased miR-10b expression and knockdown of NPM1-mA decreased miR-10b expression.

Considering the high levels of miR-10b in NPM1-mutated leukemia cells, we examined the impact of miR-10b on NPM1-mA mediated differentiation inhibition. First we found that miR-10b was downregulated as leukemia cells transition into mature monocytes. A possible explanation is that certain transcription factors such as PU.1 are activated specifically during differentiation progression [35, 36]. Hypermethylation in the CpG islands upstream of miR-10b gene may be another element for miR-10b downregulation [37, 38]. Next we found that enforced expression of miR-10b inhibited myeloid differentiation while suppression of miR-10b promoted myeloid differentiation. Furthermore, we performed a rescue assay and found that forced expression of miR-10b could reverse NPM1-mA knockdown-mediated enhancement of myeloid differentiation. These data indicated a potential role for miR-10b in NPM1-mA mediated differentiation inhibition. MiRNAs commonly supress large sets of genes. In the present study, we identified KLF4 as a direct target of miR-10b in leukemia cells. Similarly, Tian *et al.* [21] reported that KLF4 was a target of miR-10b in human esophageal cancer. Of note, low levels of KLF4 were found in NPM1-mutated AML cohorts. As a myeloid differentiation marker, KLF4 regulates terminal differentiation of monocytes and macrophages [39, 40]. Recent findings have shown a handful of novel miRNA-mRNA regulation (MMR) pairs in NPM1-mutated AML [41]. Interestingly, MMR could be modulated by *NPM1* mutation. Indeed, *NPM1* mutation modulated MMR was proposed as a novel prognostic marker in AML [42]. In our current study, we observed distinct miR-10b and KLF4 mRNA signatures in NPM1-mutated AML. Additionally, myeloid differentiation involves many regulators, including several transcription factors such as PU.1 [43], C/EPBα[44], and signal transduction pathways such as RAF-1/MEK/ERK [45]. Thus, other molecular mechanisms should be explored in the regulation of NPM1-mutated AML cells differentiation. Our observations warrant further research on the effects of NPM1 mutations on myeloid differentiation, including clinical studies and experiments with animal models. Further studies are needed to elucidate the specific effects of NPM1 mutations on miR-10b expression and the effects of elevated KLF4 expression on cell differentiation in NPM1-mutated AML cells. Lastly, it would be interesting to determine whether NPM1 mutants can regulate the dynamic interaction between miR-10b and *KLF4* mRNA.

In conclusion, our data provided direct evidence that NPM1-mA inhibited myeloid differentiation through the miR-10b/KLF4 axis. Therefore, our results highlight miR-10b and KLF4 as potential therapeutic targets and novel prognostic markers for NPM1-mA positive leukemia.

## MATERIALS AND METHODS

### Patient samples

Bone marrow (BM) samples from 12 AML patients newly diagnosed through cytomorphology, cytogenetic and molecular genetic analysis, including eight NPM1-wild type (NPM1-wt) and four NPM1-mutation type A (NPM1-mA), were obtained from Southwest Hospital of the Third Military Medical University. All patients were classified according to the French-American-British (FAB) criteria. Informed consent in accordance with the Declaration of Helsinki was obtained from the individuals examined, and the study was approved by the Institutional Review Board of the Southwest Hospital of the Third Military Medical University. Samples were enriched for mononuclear cells by Ficoll gradient purification. Following enrichment, the percentage of leukemic cells was at least 80%. The isolated mononuclear cells were used for *NPM1-mA*, *miR-10b* and *KLF4* relative expression analyses. Details of the clinical characteristics of patients are provided in Table 1.

**Table 1:** Clinical characteristics of newly diagnosed AML patients

Characteristics	NPM1-wt (*n* = 8)	NPM1-mut (*n* = 4)
Sex, Male/Female	3/5	2/2
Median age, y (range)	38.5 (26–58)	53 (43–72)
Median WBC, × 10^9^/L	17.5 (0.7–280)	23.5 (1.3–295)
Median platelets, × 10^9^/L	54.7 (8.0–455.0)	61.4 (10.0–655.0)
FAB classification		
M1	1	2
M2	1	0
M3	4	0
M4	2	1
M5	0	1
Karyotype		
Normal	1	4
t (8;21)	1	0
t (15;17)	4	0
inv (16)	2	0
Gene mutations		
*FLT3/ITD* (+/−)	2/6	2/2
*WT1* (+/−)	3/5	3/1
*KIT* (+/−)	1/7	3/1

Note: AML: acute myeloid leukemia; NPM1-wt: NPM1-wild type; NPM1-mut: NPM1- mutation; WBC: white blood cell; FAB classification: French-American-British classification, a classification of acute leukemia produced by three-nation joint collaboration.

### Cell culture and differentiation induction

Human myeloid leukemia cell lines KG-1a, HL60, OCI-AML3 (harboring NPM1-mA), THP-1 and K562 were maintained in RPMI-1640 medium (Gibco, USA) supplemented with 10% fetal bovine serum (FBS, USA) and 1% penicillin-streptomycin (Sangon biotech, China). Human embryonic kidney cells HEK293T were cultured in DMEM supplemented with 10% FBS and 1% penicillin-streptomycin. Cultured cells were incubated in a humidity chamber (Thermo Fisher Scientific, USA) containing 5% CO_2_ at 37°C. OCI-AML3 cell lines were obtained from Deutsche Sammlung von Mikroorganismen und Zellkulturen GmbH (DSMZ, Germany); other cell lines were obtained from American Type Culture Collection (ATCC, USA). For differentiation induction, 1, 25-(OH)_2_Vitamin D3 (Vitamin D3, Sigma, USA) was added to HL60 cells at a final concentration of 100 nM, and Pyromellitic acid (PMA, Beyotime, China) was added to THP-1 and OCI-AML3 cells at a final concentration of 50 nM.

### Reverse transcription PCR and quantitative real-time PCR

RNAs were isolated from the harvested cells by TRIzol (Takara, Japan) and transcribed into cDNA using PrimeScript^™^ RT reagent Kit (Takara, Japan). The resulting cDNAs were used for detection of *NPM1-mA* in primary AML blasts and cell lines by reverse transcription PCR (RT-PCR) or used for *KLF4* expression in primary AML blasts by quantitative real-time PCR (qRT-PCR) using SYBR^®^ Premix Ex Taq^™^ II (Takara, Japan) kit on a CFX Connect^™^ real-time system (Bio-Rad, USA). Cycling conditions were 5 min at 94°C for the initial denaturation, and amplification was performed with 40 cycles of 30 s at 94°C, 30 s at 56°C (for *NPM1-mA*) or 59.1°C (for *KLF4*), 50 s at 72°C, and finally 10 min at 72°C for extension. OCI-AML3 cDNA was used as a positive control for *NPM1-mA* gene amplification. For miR-10b detection, small RNA was extracted from cells by RNAiso (Takara, Japan) and transcribed into cDNA with specific RT primers. The resulting cDNAs were then used as templates for qRT –PCR. MiR-10b expression was normalized to U6. Cycling conditions were 30 s at 94°C for initial denaturation, and 40 cycles for 10 s at 94°C, 20 s at 55.8°C, 30 s at 72°C. The primers used for RT-PCR and qRT-PCR are listed in Table 2. Three independent experiments were performed in triplicate.

**Table 2:** Sequences used for RT-PCR and qRT-PCR

Genes	Sequence (5′–3′)
*NPM1-mA*	F: TGGAGGTGGTAGCAAGGTTC
	R: CTTCCTCCACTGC CAGACAGA
*KLF4*	F: TACCAAGAGCTCATGCCACC
	R: CGCGTAATCACAAGTGTGGG
*β-actin*	F: TAGTTGCGTTACACCCTTTCTTG
	R: TGCTGTCACCTTCACCGTTC
*miR-10b*	RT: GTCGTATCCAGTGCGTGTCGTGGAGTCGGCAATT GCACTGGATACGACCACAAA
	F: GGATACCCTGTAGAACCGAA
	R: CAGTGCGTGTCGTGGAGT
*U6*	RT: CGCTTCACGAATTTGCGT
	F: CTCGCTTCGGCAGCACA
	R: AACGCTTCACGAATTTGCGT

Note: F stands for forward; R stands for reverse; RT stands for reverse transcription.

### Western blot

Following the designated treatments, cells were harvested and lysed in ice-cold RIPA lysis buffer supplemented with protease inhibitor cocktail (Roche, Switzerland). Nuclear and cytoplasmic fractions were isolated using Nuclear and Cytoplasmic Protein Extraction Kit (Beyotime, China) according to the manufacturer's protocol. Equal amounts of proteins were separated by SDS-PAGE and transferred to polyvinylidene difluoride (PVDF) membranes. Primary antibodies against the following proteins were used: Rabbit monoclonal antibody NPM1 (Santa, 1:500), Rabbit polyclonal antibody against the mutated NPM1 (Abcam, 1:1,000), Rabbit polyclonal antibody GFP (Abcam, 1:1,000), Rabbit monoclonal antibody H2A.X (Cell Signaling Technology, 1:1,000), Mouse monoclonal antibody β-actin (ZSGB-Bio, Beijing, 1:1,000), Mouse monoclonal antibody KLF4 (Santa, 1:500). Horseradish peroxidase-conjugated secondary antibodies were also used. Signals were detected using an ECL (enhanced chemiluminescence) kit (Millipore, USA).

### Immunocytochemistry

THP-1 cells were washed with phosphate-buffered saline (PBS) and cytospun onto coverslips at 500 g for 5 min, fixed with 4% paraformaldehyde for 20 min at room temperature, and permeabilized with 0.1% Triton for 15 min at room temperature. Following blocking with 1% bovine serum albumin in PBS for 30 min, cells were immunostained with anti-NPM1-mA antibody overnight at 4°C. The primary antibody was revealed using the immunoalkaline phosphatase APAAP technique. Cells were counterstained with hematoxylin and mounted in neutral gum and analyzed using a bright field microscope.

### Cell transfection and infection

pEGFPC1-NPM1-mA, pEGFPC1-NPM1-wt and empty vector pEGFPC1, kindly provided by Dr. B. Falini (Institute of Hematology, University of Perugia, Perugia, Italy), were transfected into THP-1 cells using the Lipofectamine^™^ 2000 (Invitrogen, USA) reagent according to the manufacturer's instructions, which were designated as “NPM1-mA”, “NPM1-wt” and “Vector” groups, respectively. THP-1 cells without transfection were named as “Mock” group. Stable cell lines were selected with 400 μg/mL G418 (Invitrogen, USA) and the transfection efficiency was confirmed by western blot. ShRNA targeting *NPM1* (5′-AGCAAGGTTCCACAGAAAA-3′) and scramble lentiviral vectors were purchased from Gene Pharma (Shanghai, China). OCI-AML3 cells were infected with lentivirus for 48 h in the presence of 5 μg/mL polybrene (Sigma, USA), followed by 2 μg/mL puromycin selection for 7 days (Sigma, USA). The puromycin-resistant cells were isolated and propagated for use in the experiments.

### Flow cytometry

Following the designated treatments, cells were harvested at the indicated time points. The cells were washed twice with phosphate-buffered saline (PBS) and resuspended in 100 μL PBS. Then the cells were incubated in 5% bovine serum albumin/PBS with phycoerythrin (PE)-conjugated anti-CD14 (4A Biotech, Beijing) in the dark at 4°C for 30 min. Cells were washed with 5% bovine serum albumin/PBS and resuspended in 200 μL PBS. The percentage of CD14-positive cells was determined using an Accuri C6 flow cytometer (BD Biosciences, USA). Three independent experiments were performed in triplicate.

### Wright-giemsa staining and morphological observation

For assessment of morphologic differentiation, THP-1, OCI-AML3 and HL60 cells were treated as indicated for 72 h. Cells were harvested and washed twice with PBS and resuspended in 200 μL PBS. Then, the cells were cytospun and stained with Wright-Giemsa stain. The slides were washed briefly with deionized water. Cellular morphology was observed by light microscopy. The percentage of morphological differentiation was determined by counting at least 200 cells on the slides. Morphologic criteria for differentiation included the presence of the following features: nuclear/cytoplasmic ratio, indentation of the nucleus, and number of vacuoles in the cytoplasm. Three independent experiments were performed in triplicate.

### Transient transfection with miRNA mimics and inhibitors

The miR-10b mimics, inhibitors and negative controls were designed and synthesized by Gene Pharma (Shanghai, China). The primers of miR-10b mimics, miR-10b inhibitors and their own scramble controls are listed in Table 3. Cells were seeded at 4 × 10^6^ cells per well in 6-well plates. MiR-10b mimics/scramble and miR-10b inhibitors/scramble were transfected into HL60 and OCI-AML3 cells using the Lipofectamine 2000 (Invitrogen, USA) reagent according to the manufacturer's instructions, respectively. After 48 h of transfection, the cells were induced toward differentiation by PMA treatment. CD14 expression and the differentiated cells were assayed as required.

**Table 3:**
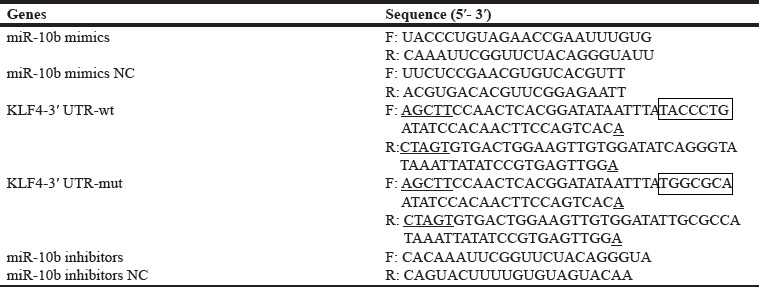
Sequences of oligonucleotides used

In rescue assays, OCI-AML3 cells stably expressing shNPM1 or empty vector were seeded at 5 × 10^6^ cells per well in 6-well plates. The cells were transfected with the miR-10b mimics or scramble, respectively. After 24 h of transfection, the cells were treated with PMA for 72 h. Finally, the cells were harvested and CD14 expression and the differentiated cells were assayed as required.

### Luciferase reporter assay

The miR-10b targets were obtained from Targetscan (http://www.targetscan.org) and Pictar (http://pictar.mdc-berlin.de/). A total of 7048 targets of miR-10b have been predicted and the evolutionarily conserved sequences present in the target mRNAs are also available. Among the targets predicted, KLF4 functioning in myeloid differentiation [24] was selected for this study. Next, KLF4-3′UTR-wild type and KLF4-3′UTR-mutation were cloned into the 3′UTR region of the firefly luciferase reporter gene in the pMIR-REPORT vector (Promega, USA), which was cleaved with *Spe* I or *Hind* III. They were designated as pMIR-KLF4-3′UTR-wt and pMIR-KLF4-3′UTR-mut, respectively. The primers for KLF4-3′UTR-wt/mut are listed in Table 3. HEK293T cells were co-transfected with either pMIR-KLF4-3′UTR-wt or pMIR-KLF4-3′UTR-mut along with miR-10b mimics or scramble using Lipofectamine 2000 (Invitrogen, USA) in a 48-well plate according to manufactures' introduction. Luciferase activity was measured using the Dual-Luciferase Reporter Assay System (Promega, USA) after 48 h of transfection. Renilla luciferase was normalized to firefly luciferase activity. All transfection assays were carried out in triplicate.

### Analysis of gene and microRNA expression profiles in patients

RNA-Seq data for gene and microRNA expression in 200 AML patients provided by TCGA database were downloaded from the UCSC Cancer Genomics Browser (https://genome-cancer.ucsc.edu). The values (normalized RNA-Seq data) represented the expression and mutation status in the gene expression matrix and the gene mutation matrix, respectively. The NPM1 mutation occurs frequently in normal karyotype AMLs and cytogenetic abnormalities are responsible for deregulation of miRNA in AML; therefore, only NK-AML cases were included in our study. Finally, *miR-10b* and *KLF4* mRNA expression data from 60 NK-AML patients were used in subsequent analyses. For statistical analysis, *miR-10b* and *KLF4* mRNA expression between the NK-AMLs with NPM1 mutation and those with no NPM1 mutation were compared via the unpaired *t* test.

### Statistical analysis

Data were presented as the mean ± SE for a minimum of three different measurements, and quantitative data from the miR-10b expression, the percentage of morphological differentiation, and CD14-positive cells were evaluated for statistical significance using the one-way ANOVA test. The relative expression of miR-10b in NPM1-mutated and NPM1-ummutated AML patients was compared using unpaired *t* test. The SPSS (Version 13.0) software and GraphPad (Prism 5) was used for statistical analyses.
